# X-ray structure of human aldo–keto reductase 1C3 in complex with a bile acid fused tetrazole inhibitor: experimental validation, molecular docking and structural analysis†

**DOI:** 10.1039/d2md00387b

**Published:** 2022-12-01

**Authors:** Maja A. Marinović, Sofija S. Bekić, Michael Kugler, Jiří Brynda, Jana Škerlová, Dušan Đ. Škorić, Pavlína Řezáčová, Edward T. Petri, Andjelka S. Ćelić

**Affiliations:** a Faculty of Sciences, Department of Biology and Ecology, University of Novi Sad Trg Dositeja Obradovića 2 21000 Novi Sad Serbia andjelka.celic@dbe.uns.ac.rs; b Faculty of Sciences, Department of Chemistry, Biochemistry and Environmental Protection, University of Novi Sad Trg Dositeja Obradovića 3 21000 Novi Sad Serbia; c Institute of Organic Chemistry and Biochemistry, The Czech Academy of Sciences Flemingovo nám. 2 Prague 16610 Czech Republic

## Abstract

Aldo–keto reductase 1C3 (AKR1C3) catalyzes the reduction of androstenedione to testosterone and reduces the effectiveness of chemotherapeutics. AKR1C3 is a target for treatment of breast and prostate cancer and AKR1C3 inhibition could be an effective adjuvant therapy in the context of leukemia and other cancers. In the present study, steroidal bile acid fused tetrazoles were screened for their ability to inhibit AKR1C3. Four C24 bile acids with C-ring fused tetrazoles were moderate to strong AKR1C3 inhibitors (37–88% inhibition), while B-ring fused tetrazoles had no effect on AKR1C3 activity. Based on a fluorescence assay in yeast cells, these four compounds displayed no affinity for estrogen receptor-α, or the androgen receptor, suggesting a lack of estrogenic or androgenic effects. A top inhibitor showed specificity for AKR1C3 over AKR1C2, and inhibited AKR1C3 with an IC_50_ of ∼7 μM. The structure of AKR1C3·NADP^+^ in complex with this C-ring fused bile acid tetrazole was determined by X-ray crystallography at 1.4 Å resolution, revealing that the C24 carboxylate is anchored to the catalytic oxyanion site (H117, Y55); meanwhile the tetrazole interacts with a tryptophan (W227) important for steroid recognition. Molecular docking predicts that all four top AKR1C3 inhibitors bind with nearly identical geometry, suggesting that C-ring bile acid fused tetrazoles represent a new class of AKR1C3 inhibitors.

## Abstract 2

Steroid bile acid fused tetrazoles were screened for ability to inhibit aldo–keto reductase 1C3 (AKR1C3), a target for cancer treatment. The X-ray structure of AKR1C3 in complex with a bile acid tetrazole inhibitor is presented.

## Graphical abstract

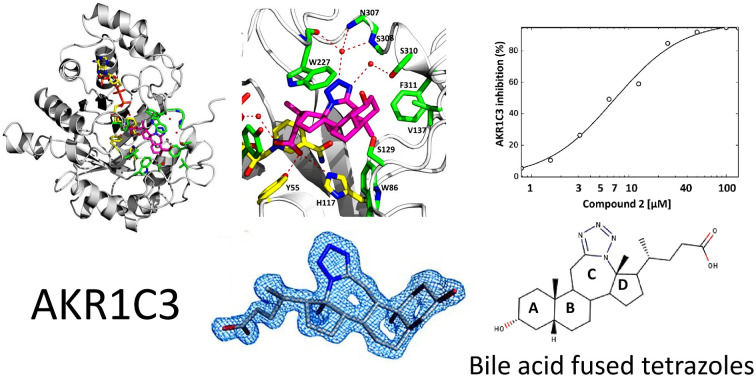

## Introduction

Human aldo–keto reductase 1C3 (AKR1C3; also known as type 5 17β-hydroxysteroid dehydrogenase, prostaglandin F synthase and 3-α-hydroxysteroid dehydrogenase type 2) is a member of the AKR superfamily of NAD(P)H-dependent oxidoreductases that catalyzes the reduction of a diverse range of carbonyl containing substrates, including exogenous compounds and therapeutic drugs.^1,2^ AKR1C3 has high sequence similarity to other AKR1C isoforms, AKR1C1, AKR1C2 and AKR1C4, which act as 20-keto, 3-keto and 17-ketosteroid hormone reductases.^1,3^ AKR1C3 functions primarily as a 17-ketosteroid reductase that catalyzes NADPH-dependent reduction of weaker steroid hormones to more potent androgens and estrogens: including the reduction of androstenedione to testosterone, 5α-androstanedione to 5α-dihydrotestosterone (DHT) and estrone to 17β-estradiol.^4^ Through its enzymatic activity, AKR1C3 regulates androgen and estrogen levels in peripheral tissue; it also indirectly affects estrogen levels by increasing the availability of testosterone for aromatase, which converts testosterone to estrogen.^4,5^ AKR1C3 is overexpressed in prostate cancer and most breast cancer types, which makes it an excellent target for the treatment of hormone-dependent cancers.^1,6^ In advanced prostate cancer, AKR1C3 expression is associated with resistance to abiraterone and enzalutamide, and treatment with an AKR1C3 inhibitor (indomethacin) restores sensitivity to these chemotherapeutics.^1,7–9^ In acute myeloid leukemia (AML), AKR1C3 reduces prostaglandin PGD_2_ to 9α,11β-prostaglandin F2, affecting cell proliferation and differentiation.^1,10–12^ Treatment with a pan-AKR1C inhibitor, medroxyprogesterone acetate (MPA), in combination with a lipid regulating drug (bezafibrate), results in growth arrest and differentiation in AML cells *in vitro* due to inhibition of PGD_2_ metabolism by AKR1C3;^1,11,13^ meanwhile treatment of AML patients with MPA/bezafibrate in a clinical trial yielded positive results.^13,14^ AKR1C enzyme activity also confers resistance to chemotherapy in T-cell acute lymphoblastic leukemia (T-ALL) and AKR1C3 inhibitors against T-ALL have been reported.^15,16^ Because of its roles in the progression of hormone-dependent and other cancers, as well as in the development of resistance to chemotherapeutics, research efforts have focused on identifying specific small-molecule inhibitors of AKR1C3, as well as pan-AKR1C inhibitors, for clinical use.^17–19^

X-ray structures of AKR1C3 in complex with various substrates and inhibitors have revealed a large ligand binding site and a significant degree of conformational plasticity among amino acid residues involved in ligand recognition.^5,12,20–23^ Because of this, and the structural similarities between AKR1C isoforms, few studies have been able to use molecular docking alone to predict the bioactivities of new compounds against AKR1C3. Thus, structure-based drug design efforts have relied on protein X-ray crystallography, resulting in crystal structures of AKR1C2 and AKR1C3 in complex with a range of ligands.^5,12,20–23^ Based on these structures, AKR1C inhibitors with carboxylic acid functional groups that interact directly with the nicotinamide ring of the NADPH cofactor and amino acid residues involved in catalysis have been characterized.^5,20,22,23^ Structures of AKR1C3 in complex with a steroidal inhibitor and of AKR1C2 in complex with a bile acid inhibitor, ursodeoxycholate, show that steroidal carboxylates could be used as a scaffold for the design of AKR1C3 inhibitors.^21,24^

Bile acids and other steroidal carboxylates are known inhibitors of AKR1C3, but display specificity for AKR1C2.^25,26^ As part of our efforts to characterize the anticancer properties of synthetic steroidal compounds, a series of heterocyclic steroidal compounds were tested as inhibitors of AKR1C3 resulting in identification of an A-ring fused pyridine derivative with an IC_50_ of ∼15 μM for AKR1C3.^27^ Because both bile acids and heterocyclic steroids have AKR1C3 inhibition properties, we became interested in testing bile acid derivatives with heterocyclic modifications as AKR1C3 inhibitors. Recently, the synthesis and chemical characterization of a series of bile acid fused tetrazoles with anticancer properties was described by members of our group.^28^ Tetrazole modifications can change the bioactivity of the parent compounds by changing the hydrogen bonding potential and introducing a carboxylic acid bioisostere.^29^ Thus, the aim of the present study was to test the potential of this series of bile acid fused tetrazoles as AKR1C3 inhibitors using enzymatic assays *in vitro*. The structural basis of AKR1C3 inhibition by the top ranking bile acid tetrazole was then investigated by protein X-ray crystallography and molecular docking.

## Results and discussion

### Identification of C24 bile acid C-ring fused tetrazoles as inhibitors of AKR1C3

**Fig. 1:**
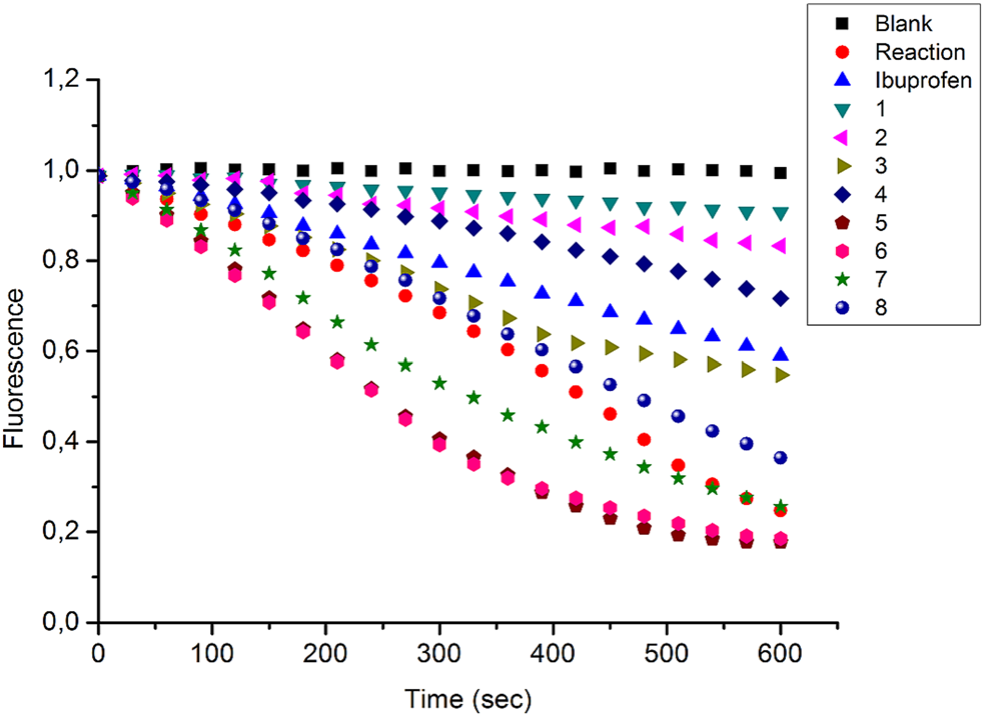
Change in NADPH fluorescence during reduction of 9,10-phenanthrenequinone by AKR1C3 in the presence or absence of bile acid fused tetrazoles 1–8. Reactions were conducted in the absence (reaction) or presence of compounds 1–8 (33.3 μM), or ibuprofen (33.3 μM), included as a positive control AKR1C3 inhibitor. A negative control reaction (blank) was conducted in the absence of AKR1C3 enzyme. Data shown represent the mean of three independent experiments.

In the present study, a series of bile acid derivatives (1–8) with a tetrazole group fused to either the B- or C-ring of the steroidal core were tested for their ability to inhibit the reduction of 9,10-phenanthrenequinone (PQ), a general AKR1C substrate, by human AKR1C3.^28^ Initial screening of the effect of 1–8 on AKR1C3 enzymatic activity was conducted using a standard assay, by monitoring NADPH consumption during the reaction using fluorescence spectroscopy (excitation 340 nm, emission 460 nm) (Fig. 1 and Table 1).^26,27,30,31^

**Table:** AKR1C3 inhibition potential of bile acid tetrazole compounds (1–8). Calculated values for percent inhibition (% inhibition) of AKR1C3 reduction of 9,10-phenanthrenequinone by bile acid fused tetrazole compounds 1–8 are shown (see also Fig. 1). NI = no inhibition

	Compound	% inhibition AKR1C3
1	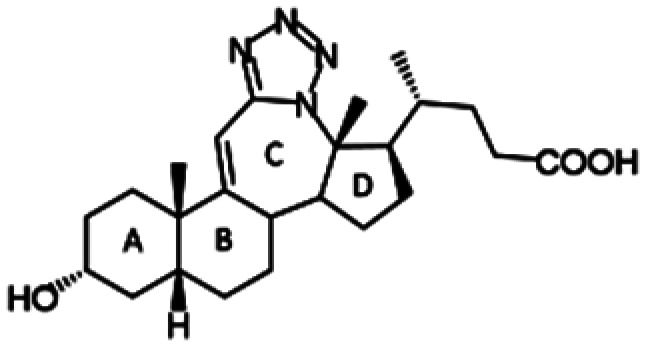	88.4%
2	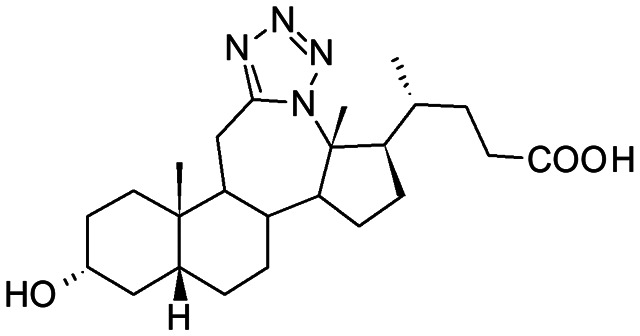	77.93%
3	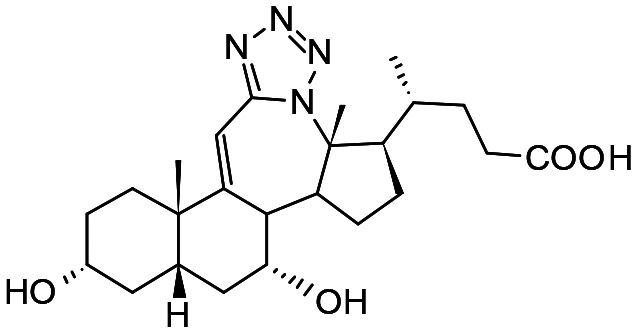	37.60%
4	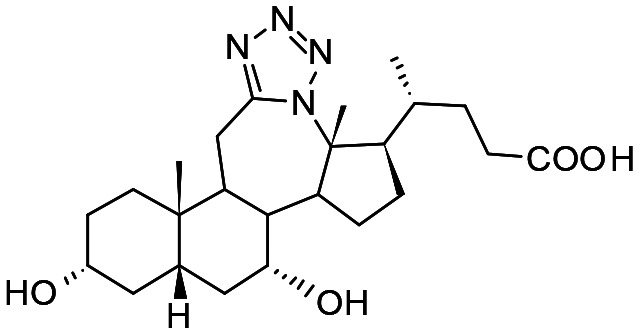	64.83%
5	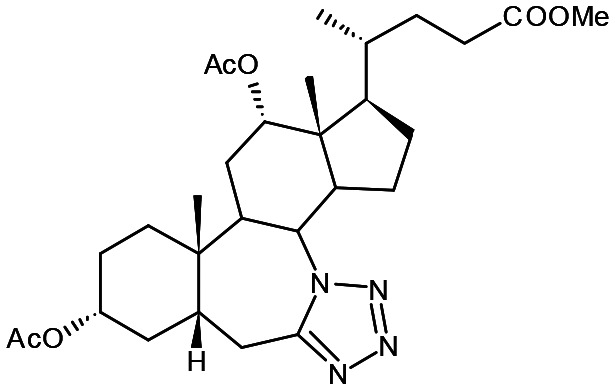	NI
6	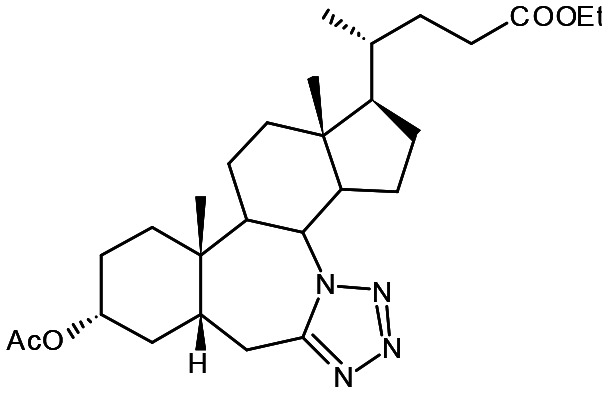	NI
7	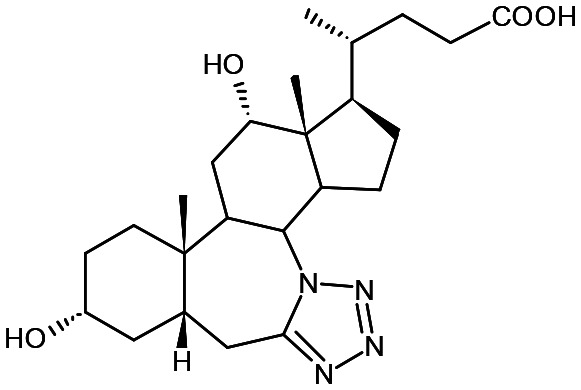	NI
8	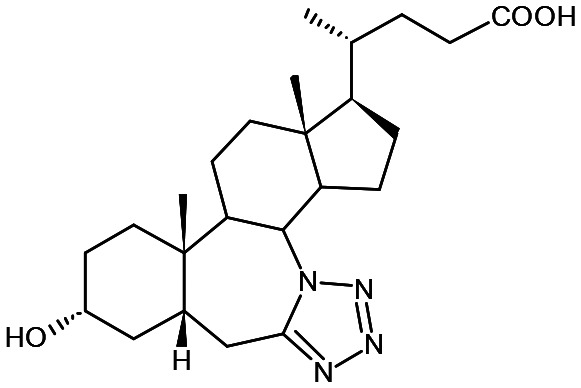	14.17%

Based on this *in vitro* enzymatic screen, compounds 1–4 and 8 were identified as potential inhibitors of AKR1C3 (Fig. 1 and Table 1). Bile acid derivatives 1, 2 and 4 with a C-ring fused tetrazole reduced AKR1C3 activity by more than 50%, with compounds 1 (88%) and 2 (78%) displaying the strongest inhibition potential. Under the same assay conditions, ibuprofen inhibited 46% of AKR1C3 activity, similar to reported values in the literature.^20,32^ In contrast, compounds with a B-ring fused tetrazole group had either little (8, 14% inhibition) or no effect (5, 6 and 7) on AKR1C3 reduction activity. Although the number of compounds tested in the present study is small, these results do suggest that the location of the fused tetrazole on the steroidal core may be important for AKR1C3 inhibition. Comparison of a matched pair of compounds that differ only in the location of the fused tetrazole highlights this trend, where compound 2 (∼78%) with a C-ring tetrazole is a stronger inhibitor than otherwise identical compound 8 (∼14%) with a B-ring tetrazole. In summary, based on this preliminary *in vitro* enzymatic assay, C24 bile acid derivatives with a C-ring fused tetrazole could represent a starting point for the design of a new class of AKR1C3 inhibitors.

**Fig. 2:**
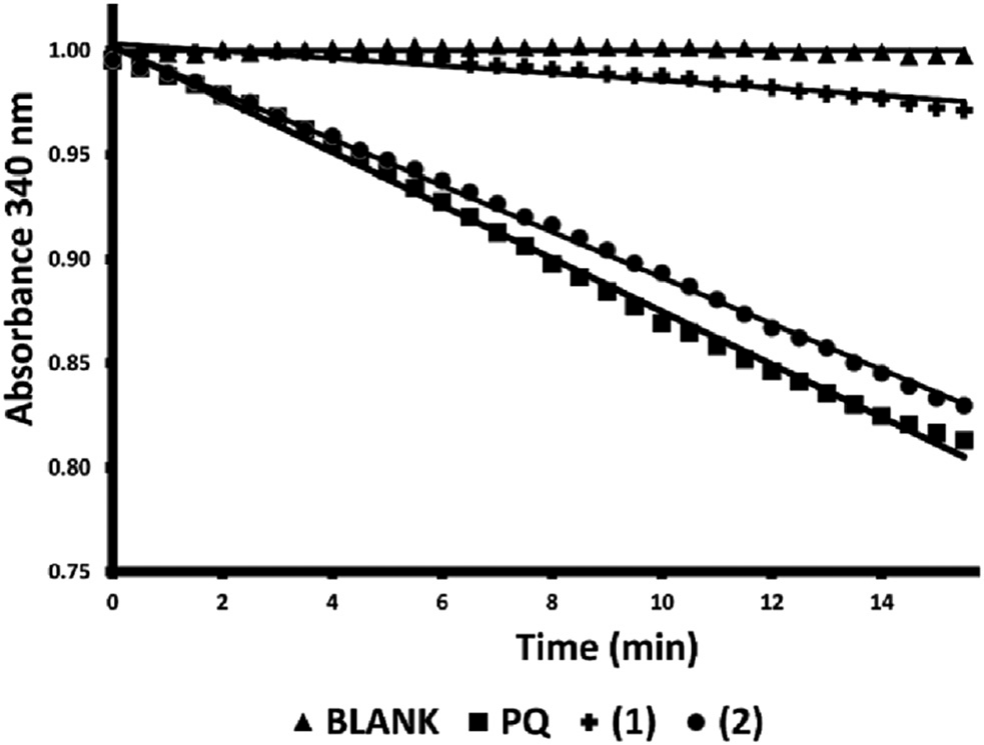
Effect of bile acid tetrazole compounds 1–2 on AKR1C2 reduction of 9,10-phenanthrenequinone (PQ). AKR1C2 activity was measured by monitoring the decrease in NADPH absorbance at 340 nm over time. Reduction of 9,10-phenanthrenequinone by AKR1C2 was measured in the absence (PQ) or presence of test compounds (1–2) at a final concentration of 40 μM. Data shown represent the mean of three experiments, fit by linear regression.

Because AKR1C2 may reduce androgen levels by catalyzing the reduction of 5α-dihydrotestosterone to 3α-androstanediol, off-target inhibition of AKR1C2 is considered to be an undesirable side effect of AKR1C3 inhibitors intended for use against hormone-dependent breast or prostate cancers.^1,4^ Thus the top two compounds (1 and 2) identified above as AKR1C3 inhibitors based on the NADPH fluorescence assay were tested for their ability to inhibit the closely related isoform, AKR1C2, using a standard spectroscopic assay as previously described.^26,27^ The effect of compounds 1 and 2 on the reduction of PQ by the AKR1C2 isoform was investigated by monitoring the consumption of NADPH, as measured by the change in absorption at 340 nm during the reaction (Fig. 2). Compound 1 appears to be a strong AKR1C2 inhibitor (86% inhibition), while compound 2 (13% inhibition) had little effect on AKR1C2 activity. In summary, compound 1 may represent a starting point for the development of pan-AKR1C inhibitors, while compound 2 appears to be a more specific inhibitor of AKR1C3.

**Fig. 3:**
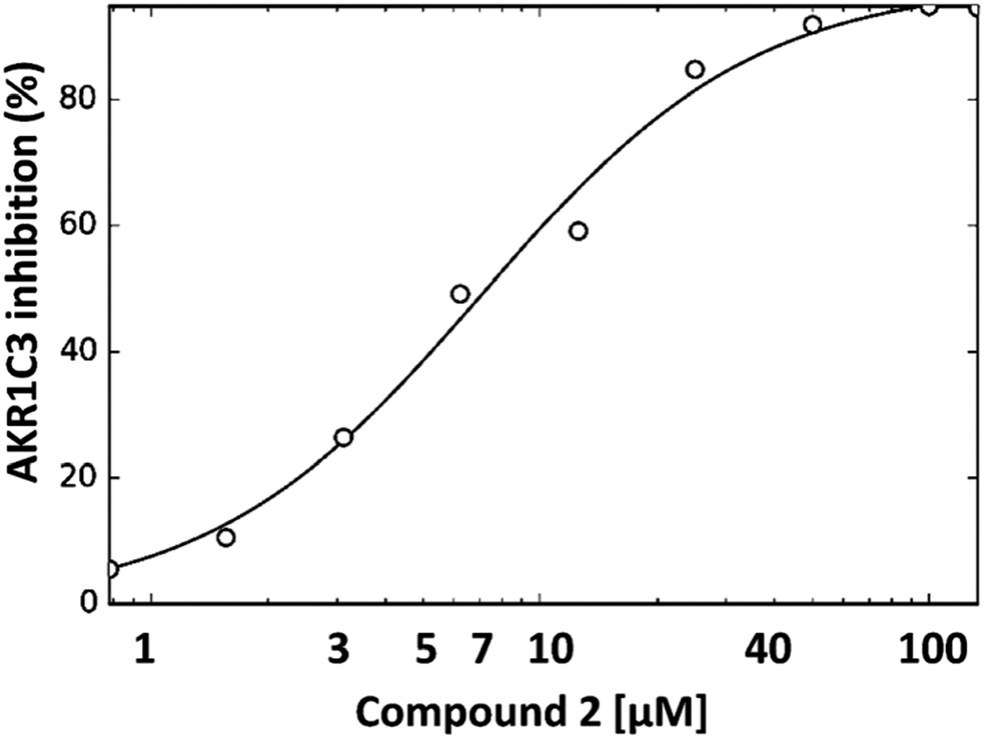
Dose-dependence of AKR1C3 inhibition by compound 2. The effect of increasing concentrations of compound 2 (0 to 100 μM) on 9,10-phenanthrenequinone reduction by AKR1C3 was tested by monitoring the change in NADPH fluorescence. Results shown represent the mean of two independent experiments.

The dose dependence of AKR1C3 inhibition by compound 2 was also determined by NADPH fluorescence spectroscopy (Fig. 3). As can be seen, under the assay conditions used, compound 2 inhibits AKR1C3 reduction of PQ in a sigmoidal, dose-dependent manner, with a resulting calculated IC_50_ value of ∼7 μM.

### Relative affinity of bile acid tetrazole compounds 1–8 for the ligand binding domains of estrogen receptor α, estrogen receptor β and the androgen receptor

**Fig. 4:**
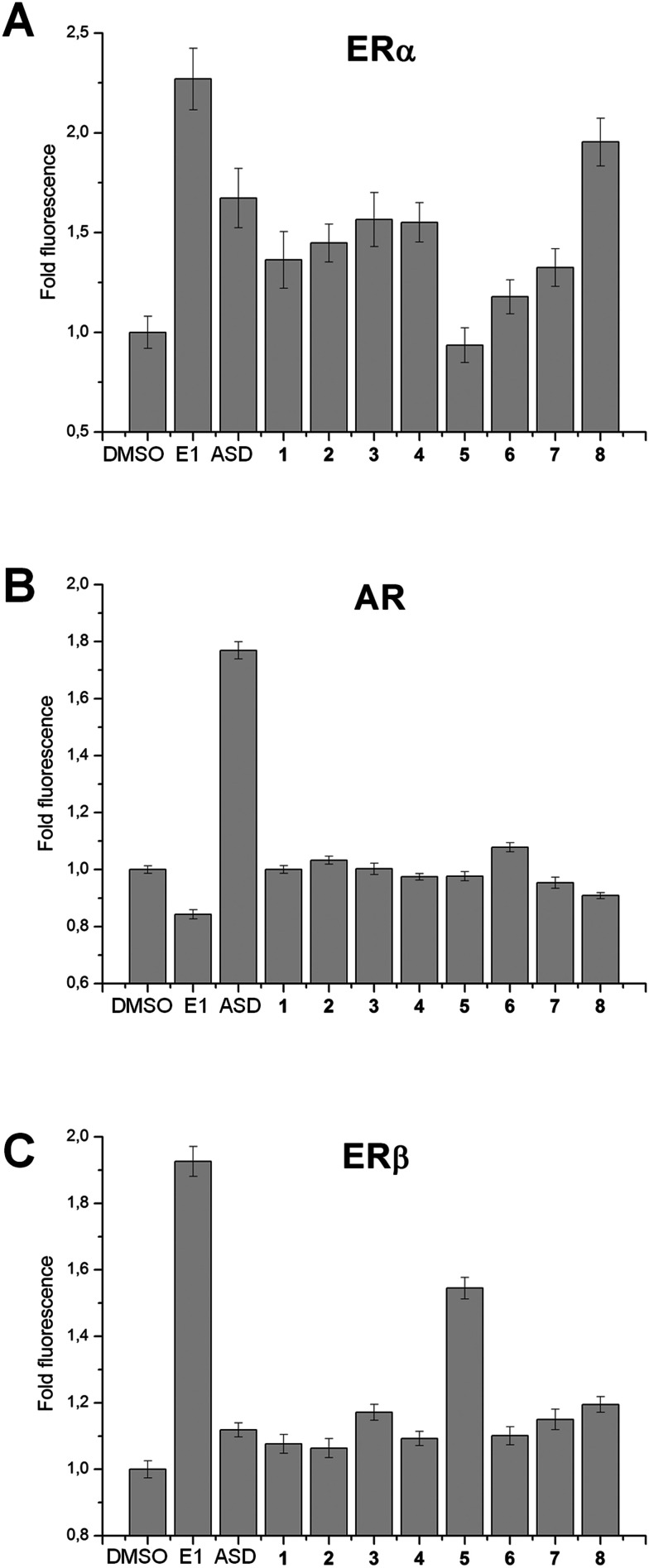
Relative affinity of compounds 1–8 for the ligand binding domains of estrogen receptor α, estrogen receptor β and the androgen receptor. Changes in fluorescence intensity in yeast cells expressing ERα- (panel A), AR- (panel B) or ERβ-LBD-YFP (panel C) upon treatment with compounds 1–8, estrone (E1), androstenedione (ASD) or vehicle (DMSO). E1 and ASD were positive and negative control ligands, respectively, for yeast cells expressing ERα- and ERβ-LBD-YFP; meanwhile ASD and E1 were positive and negative control ligands, respectively, for yeast cells expressing AR-LBD-YFP.

Compounds intended for use as treatment against hormone-dependent cancers such as breast or prostate cancer should not activate estrogen or androgen signaling pathways. To control this, the relative affinities of 1–8 for the ligand binding domains (LBD) of estrogen receptor α (ERα-LBD), estrogen receptor β (ERβ-LBD) and the androgen receptor (AR-LBD) were assessed using yeast cells expressing ERα-, ERβ- or AR-LBD fused in frame with yellow fluorescent protein (YFP), as previously described.^33–35^ Briefly, yeast cells expressing ERα-, ERβ- or AR-LBD-YFP display a dose-dependent increase in YFP fluorescence upon treatment with the appropriate cognate steroidal hormone.^34^ In the present study, estrone (E1) and androstenedione (ASD) were used as positive and negative control steroidal ligands, respectively, for yeast cells expressing ERα- and ERβ-LBD-YFP; meanwhile ASD and E1 were positive and negative control ligands, respectively, for yeast cells expressing AR-LBD-YFP. As can be seen in Fig. 4, strong fold fluorescence changes were induced upon treatment with positive control steroidal ligands for each receptor. In addition, based on this assay, compound 8 appears to have moderate affinity for ERα-LBD, while compound 5 has moderate affinity for ERβ-LBD. This assay only measures relative ligand binding affinity and cannot determine if a compound is an agonist or antagonist of the estrogen or androgen receptor. Further studies are needed to determine if 5 or 8 could have pro- or anti-estrogenic effects. Importantly, none of the top ranking AKR1C3 inhibitors identified in the present study (compounds 1–4) displayed any affinity for the LBD of ERα, ERβ or AR, suggesting a lack of estrogenic or androgenic potential.

### Crystal structure of the AKR1C3·NADP^+^·(2) ternary complex

To determine the structural basis of inhibition of AKR1C3 by bile acid fused tetrazole compound 2, we determined the X-ray structure of AKR1C3 in complex with NADP^+^ and compound 2, AKR1C3·NADP^+^·(2), at 1.4 Å resolution (PDB code: 8BBS). For data collection and refinement statistics, see Table 2.

Data collection and refinement statistics for the crystal structure of AKR1C3–NADP^+^ in complex with compound 2

**Table:** 

Data collection statistics 8BBS	
Space group	*P*1
Cell parameters *a*, *b*, *c* [Å]; *a*, *β*, *γ* [°]	39.46, 51.41, 77.8877.26, 86.74, 77.63
Wavelength [Å]	0.9184
Resolution [Å]	50.0–1.4 (1.48–1.40)
Unique reflections	107 028 (18 613)
Multiplicity	3.6 (3.6)
Completeness [%]	93.1 (92.7)
*R*_meas_ [%]^a^	6.8 (67.9)
CC_(1/2)_ [%]^b^	99.9 (82.8)
Average *I*/*σ*(*I*)	12.5 (2.1)
Wilson *B* [Å^2^]^c^	21.7

**Table:** 

Refinement statistics	
Resolution range [Å]	45.93–1.40 (1.44–1.40)
No. of reflections in working set	105 499 (7795)
No. of reflections in test set	1533 (113)
*R* value [%]^d^	15.5 (26.7)
*R*-Free value [%]^e^	17.8 (25.8)
RMSD deviation from ideal bond length [Å]	0.011
RMSD deviation from ideal bond angle [°]	1.743
Number of protein atoms	5345
Number of water molecules	728
Number of other non-protein atoms	167
Mean *B* value [Å^2^]	17.31
Residues in Ramachandran favored regions [%]^f^	98.2
Residues in Ramachandran allowed regions [%]^f^	100

a *R*_meas_ defined by Diederichs and Karplus.^36^

b CC_(1/2)_ is Pearson's correlation coefficient determined on the data set randomly split in half.^37^

c Wilson *B* by the Sfcheck program from the CCP4 suite.^38^

d *R*-Value = ||*F*_o_| − |*F*_c_||/|*F*_o_|, where *F*_o_ and *F*_c_ are the observed and calculated structure factors, respectively.

e *R*_free_ is equivalent to the *R*-value but is calculated for reflections in a test set, chosen at random and omitted from the refinement process.^39^

f As determined by MolProbity.^40^

**Fig. 5:**
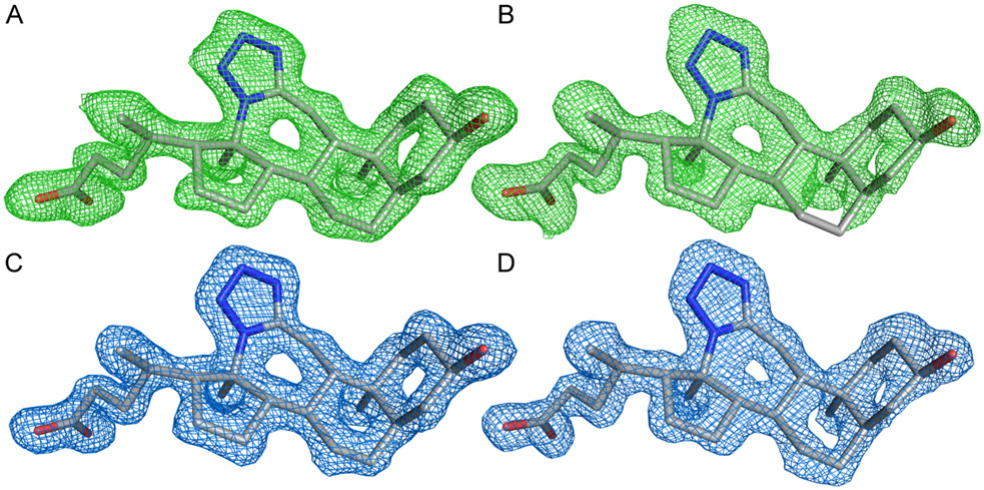
Electron density maps for compound 2. The *F*_o_ − *F*_c_ difference electron density maps for compound 2 calculated before ligand modeling are shown as a green mesh contoured at 2*σ* in panels A and B for chains A and B, respectively. Final refined 2*F*_o_ − *F*_c_ electron density maps contoured at 1*σ* (blue mesh) are shown in panels C and D for compound 2 in active sites A and B, respectively. The final refined coordinates of the ligands are shown in all panels for comparison.

The asymmetric unit comprised two molecules of AKR1C3 with clear electron density for amino acids 6–323 (chain A) and 6–319 (chain B) and the NADP^+^ cofactor. The quality of the electron density suggested a higher flexibility of the region between residues 125 and 137 and especially the C-terminus, starting from residue 307. Monomer A and monomer B have nearly identical geometries, and can be superimposed with an RMSD of 0.174 Å. The electron density corresponding to the 3D structure of compound 2 was clearly visible in the AKR1C3 active site, in both monomer A and monomer B of the asymmetric unit (see Fig. 5) and compound 2 was modeled with full occupancy in both active sites. The position and geometry of (2), NADP^+^, and residues in the ligand binding site are nearly identical between monomers A and B, with the exception of the alternative conformations of N307. Chain A will be used for the description of ligand binding, as the electron density map of the C-terminus was slightly better defined than in chain B.

**Fig. 6:**
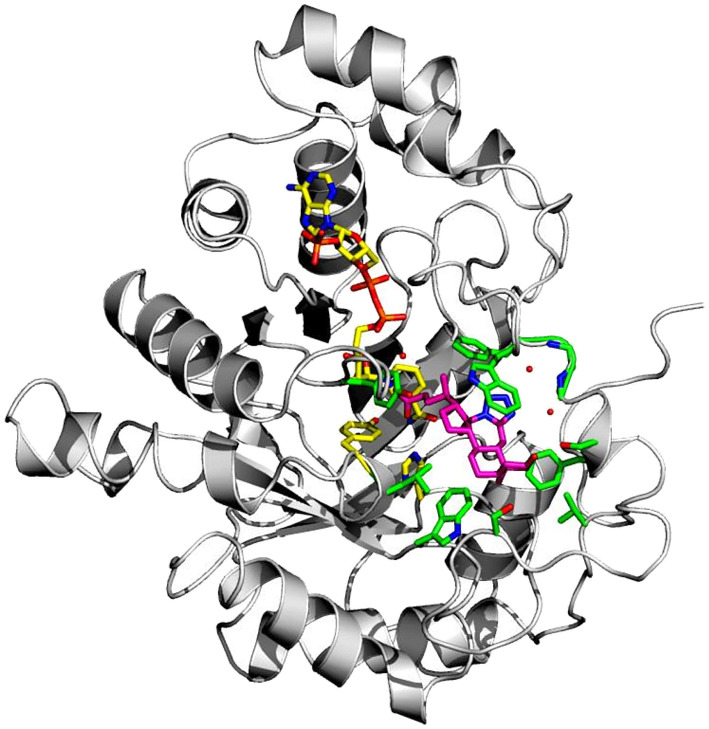
X-ray crystal structure of AKR1C3 in complex with NADP^+^ and bile acid tetrazole (2). Overall structure of AKR1C3·NADP^+^·(2), showing compound 2 (magenta sticks) bound near the NADP^+^ cofactor and catalytic residues Y55 and H117 (yellow sticks). Amino acid residues within 4 Å of compound 2 are shown as green sticks. Three water molecules that mediate hydrogen bonding between compound 2 and the AKR1C3 main chain or NADP^+^ cofactor are shown as small red spheres.

To date, there are 52 X-ray structures of AKR1C3 known in complex with various ligands and inhibitors.^19,41^ The overall structure of AKR1C3·NADP^+^·(2) is globally similar to other AKR1C3 structures, which all contain a characteristic (α/β) 8 – barrel structure, and a highly conserved NADPH cofactor binding site (Fig. 6).^3,41^ However, comparison of known AKR1C3 structures shows that the conformations of amino acid side chains and supporting loop regions involved in ligand binding are dependent on the physico-chemical properties of the bound ligand.^3^

**Fig. 7:**
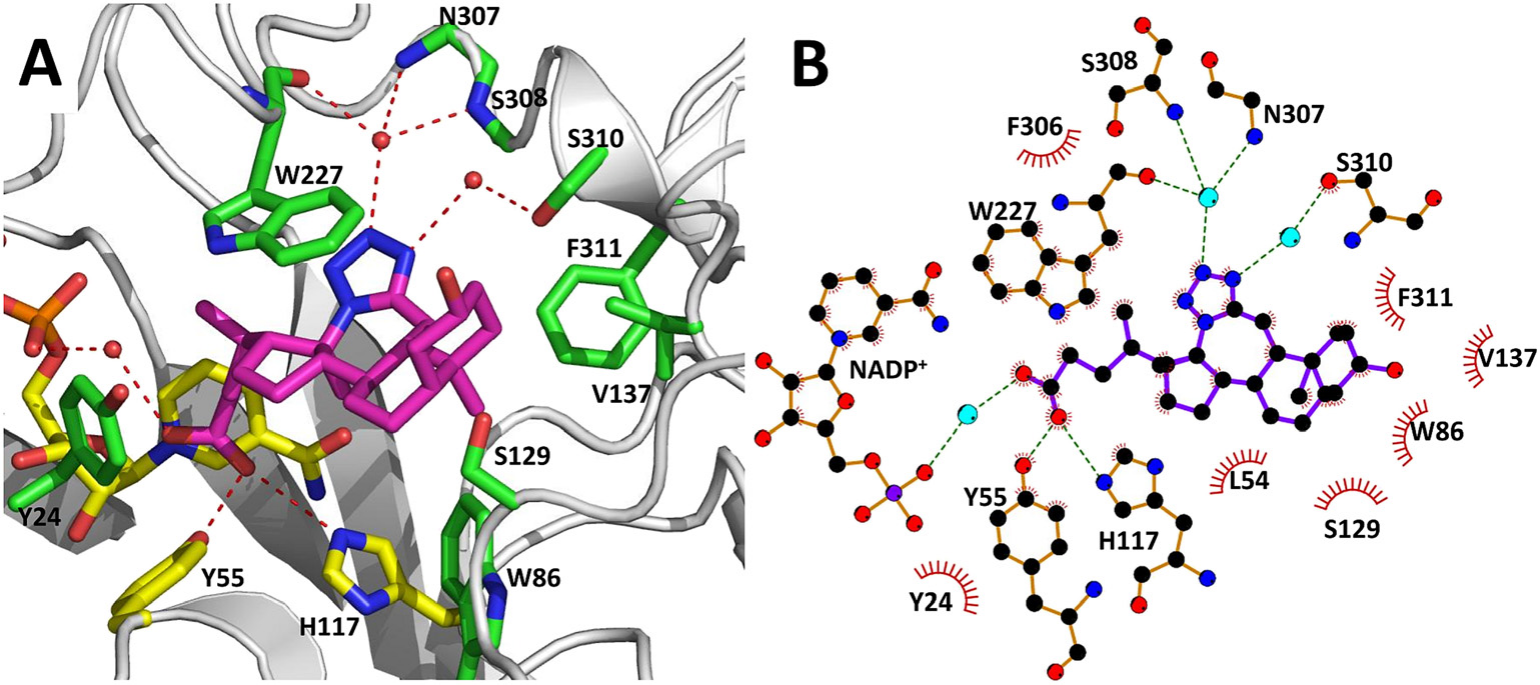
Intermolecular interactions between compound 2 and the AKR1C3 active site. Panel A shows a three-dimensional interaction network in the active site, while in panel B, a two-dimensional diagram of the same intermolecular interactions was automatically generated using the program LigPlot. Compound 2 (magenta) interacts with AKR1C3 residues corresponding to the steroid channel (W227); oxyanion site (Y55, H117, NADP^+^, yellow sticks); SP1 (F306); SP2 (W86, S129, W227, F311); and SP3 (Y24). Hydrogen bonds are depicted as dashed lines. Three waters that interact with compound 2 are shown as red spheres. Water-mediated hydrogen bonds were detected between the C-ring tetrazole and the following: main chain nitrogen atoms of N307, S308; the side chain hydroxyl of S310; and the main chain carbonyl of W227. A water-mediated hydrogen bond between the C24 carboxylate and NADP^+^ was also detected.

The AKR1C3 active site contains a steroid binding channel, an oxyanion site, and three subpockets.^3,41^ Gatekeeper residues (W227 and L54) in the steroid channel determine substrate orientation, while the oxyanion site, consisting of the nicotinamide ring from the NAD(P)H cofactor and catalytic residues H117 and Y55, anchors ketone, carboxyl or alcohol containing substrates in proximity to the cofactor for enzymatic reactions.^3,20^ In AKR1C3, ligand recognition occurs *via* interactions with one or more subpockets (SP1, SP2 or SP3), which are involved in ligand binding to varying degrees in all known AKR1C3 structures: SP1 (S118, N167, F306, F311 and Y319); SP2 (W86, S129, W227 and F311) or SP3 (Y24, E192, S217, S221, Q222, Y305 and F306).^3,41^ Note that F311 is located at the interface between SP1 and SP2 and can be in contact with ligands in SP1 or SP2. Overall, binding of compound 2 to AKR1C3 relies on hydrophobic contacts between its steroidal core and residues lining SP2, including W86, S129, W227 and F311, with support from Y24 in SP3 and F306 in SP1 (Fig. 7). To help visualize these contacts, protein–ligand interactions were projected in two dimensions using the program LigPlot.^42,43^ Compound 2 is one of only a few AKR1C3 inhibitors shown to interact strongly with SP2, suggesting that this could be exploited in the future for design of molecules with enhanced target specificity. In addition, the C24 carboxylate group is strongly anchored to the oxyanion site *via* hydrogen bonding at H117 and Y55, and a water-mediated hydrogen bond with NADP^+^; meanwhile the C-ring fused tetrazole interacts with W227 and forms potential water-mediated hydrogen bonds to the main chain nitrogen atoms of N307, S308 and side chain hydroxyl of S310, as well as the main chain carbonyl of W227 (Fig. 7).

**Fig. 8:**
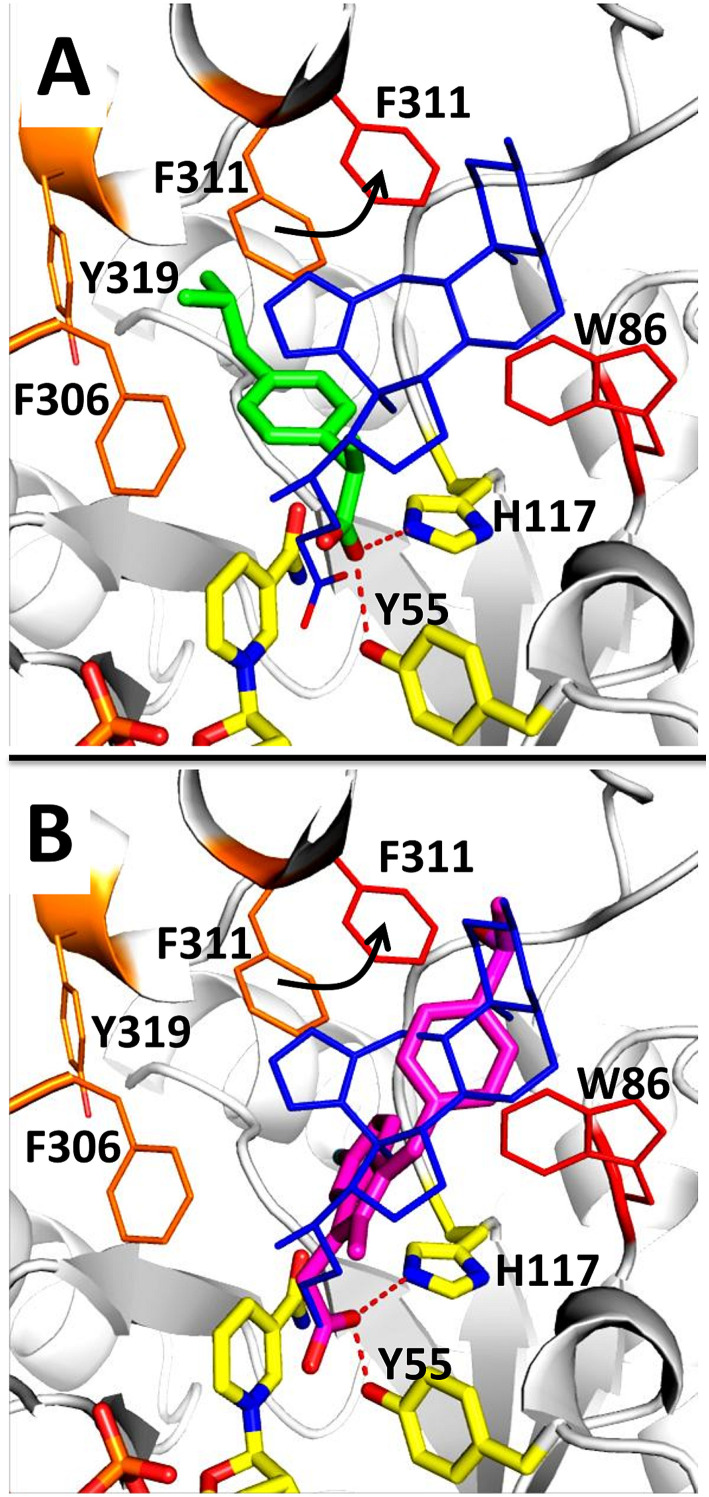
Comparison of the crystal structure of AKR1C3·NADP^+^·(2) with the structures of AKR1C3 in complex with ibuprofen (panel A) or sulindac (panel B). Compound 2 is shown as blue lines in comparison with ibuprofen (green sticks) or sulindac (magenta sticks). AKR1C3 residues comprising SP1 (F306, F311, Y319) that are in contact with ibuprofen are shown in orange, and residues comprising SP2 (W86 and F311) that are in contact with compound 2 are shown in red. Movement of F311 from an SP1 position in complex with ibuprofen, to an SP2 position in complex with compound 2 is shown as a black arrow. The oxyanion site (NADP^+^, H117, Y55) is shown as yellow sticks. SP2 residue W227 lies above the plane of compound 2 and could not be shown for clarity.

To investigate the structural basis of AKR1C3 inhibition by (2), the structure of AKR1C3 in complex with (2) was compared with the structure of AKR1C3 in complex with other known inhibitors: NSAIDs ibuprofen (3R8G) and sulindac (3R7M).^20^ AKR1C3·NADP^+^·(2) was superimposed on the structures of AKR1C3·NADP^+^·sulindac and AKR1C3·NADP^+^·ibuprofen with an RMSD of 0.250 Å and 0.240 Å, respectively, using the align function in PyMol (Fig. 8).^44^ The position and geometry of NADP^+^ was identical in all three AKR1C3 structures. Ibuprofen, sulindac and compound 2 all contain a carboxylate functional group that is involved in binding, and reported to be critical for AKR1C3 inhibition.^20,25^ Binding of the carboxylate in other NSAIDs to AKR1C enzymes has been studied by protein X-ray crystallography, showing that interactions with the oxyanion site can explain general AKR1C inhibition by NSAID compounds.^5,20^ In both AKR1C3·NADP^+^·ibuprofen and our structure, one oxygen atom from the carboxylate group interacts with the catalytically essential side chain, H117. The positions of catalytic residues, H117 and Y55, as well as those of K84 and D50, are identical between the two structures. However, the second oxygen atom in the carboxylate of (2) and sulindac is located deeper in the oxyanion hole, taking the position of a bound water in AKR1C3·NADP^+^·ibuprofen, and interacts with Y55 and the nicotinamide ring of NADP^+^. Like most AKR1C3 inhibitors, the majority of intermolecular contacts between AKR1C3 and ibuprofen involve SP1 residues.^3,41^ In contrast, both compound 2 and sulindac belong to a smaller group of inhibitors that interact with SP2.^20^ For example, in complex with ibuprofen F311 forms part of the SP1 binding site, while in complex with (2) F311 is shifted toward the SP2 site, to accommodate the tetrazole group. The steroidal core and tetrazole in (2) also form unique contacts, including water-mediated hydrogen bonding interactions. This structural comparison suggests that (2) inhibits AKR1C3 *via* a mechanism involving its C24 carboxylate, while the steroidal core and C-ring fused tetrazole provide additional unique contacts with AKR1C3 that could be exploited to improve the specificity and potency of this newly identified type of inhibitor.

### Molecular docking of compounds 1–4 into the X-ray structure of AKR1C3·NADP^+^·(2)

To further model the structural basis of AKR1C3 inhibition by bile acid fused tetrazoles, molecular docking simulations were conducted. Using the structure of AKR1C3·NADP^+^·(2) as ‘receptor’, the structural basis of AKR1C3 inhibition by compounds 1, 3 and 4 was simulated in Autodock 4 using the PyRx virtual screening tool.^26,45,46^ As a control, compound 2 was re-docked into an apo structure of AKR1C3·NADP^+^ following removal of the coordinates for (2) in a text editor. Re-docking of compound (2) in Autodock 4.2 was able to reproduce the experimental structure of AKR1C3·NADP^+^·(2) with identical geometry and a mean docking energy of −10.99 kcal mol^−1^ for the most populated cluster (representing 7 out of 10 simulation runs). Under the same simulation conditions, favorable docking energies were obtained for compounds 1, 3 and 4 (see Table 3).

**Table:** Predicted binding energies for compounds 1–4 based on molecular docking simulations in Autodock 4.2

Compound	Autodock 4.2 binding energy (kcal mol^−1^)		AKR1C3 inhibition (%)
	Maximum	Cluster mean	
2	−11.18	−10.99	77.93%
1	−10.15	−9.98	88.40%
4	−9.51	−9.51	64.83%
3	−9.59	−9.59	37.60%

Although docking simulations are not intended for ranking closely related series of chemical compounds^47^ predicted docking energies obtained here are in qualitative agreement with experimentally measured AKR1C3 inhibitory potential (% inhibition). However, docking simulations are capable of identifying and predicting the binding geometry of bioactive ligands.^47^

**Fig. 9:**
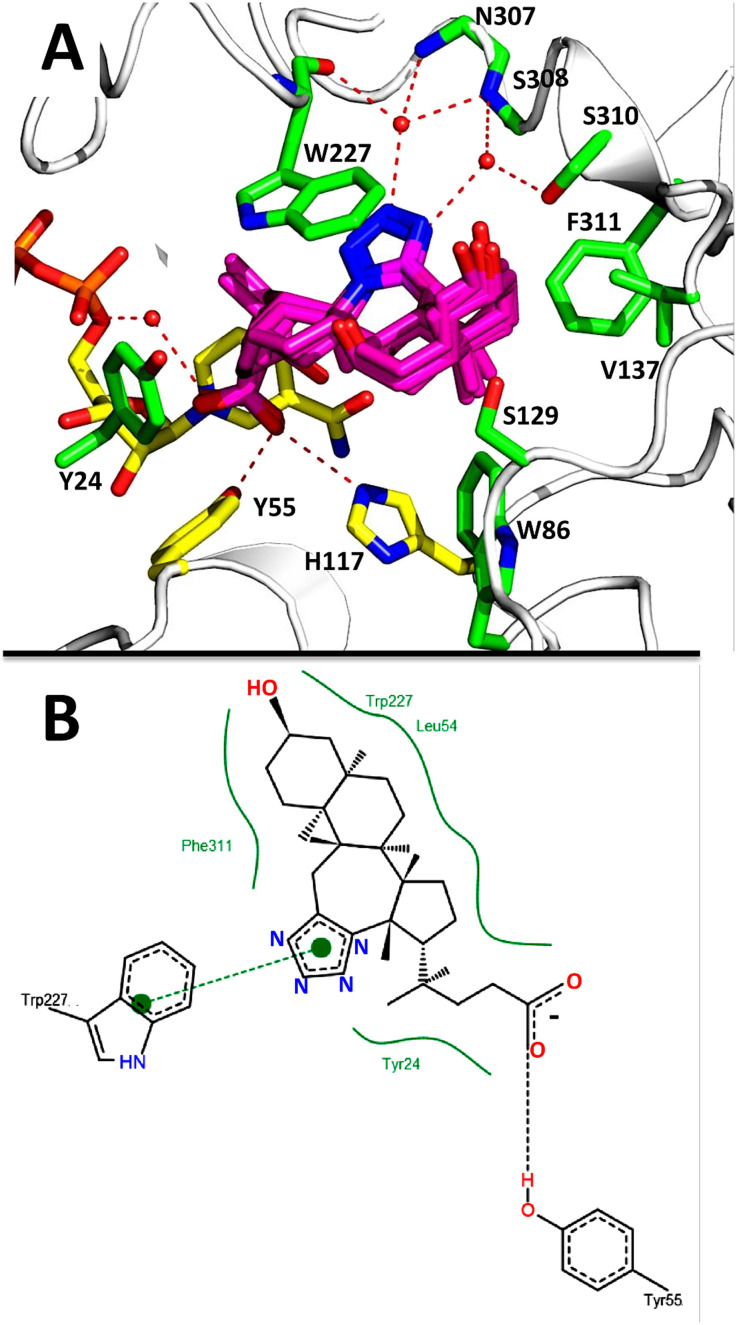
A) Molecular docking of compounds 1–4 (magenta sticks) into the active site of AKR1C3. Residues within 4 Å of compound 2 are labeled and shown as green sticks. Hydrogen bonds are shown as red dashed lines. B) Two dimensional diagram of docking results for compounds 1–4 generated using PoseView (compound 2 is shown). PoseView automatically identified several important interactions: hydrogen bonding between the C24 carboxylate of 2 and Y55 (black dashed line); hydrophobic interactions between 2 and W227, F311, L54 and Y24 (green solid lines); and a π–π interaction between the tetrazole and W227 is shown (green circles connected by a green dashed line).

Docking results for 1–4 were analyzed in PyMol, and using the program PoseView, which automatically generates a two dimensional diagram of intermolecular contacts important for a protein–ligand interaction (Fig. 9).^48^ Based on these analyses, compounds 1–4 bind AKR1C3 in a nearly identical manner, *via* intermolecular contacts formed by their C24 carboxylate, C-ring tetrazole and steroidal core atoms. PoseView also suggests that the aromatic tetrazole may form π–π interactions with the aromatic tryptophan (W227).

### Molecular docking of compound 1 onto the X-ray structure of AKR1C2 in complex with ursodeoxycholate

**Fig. 10:**
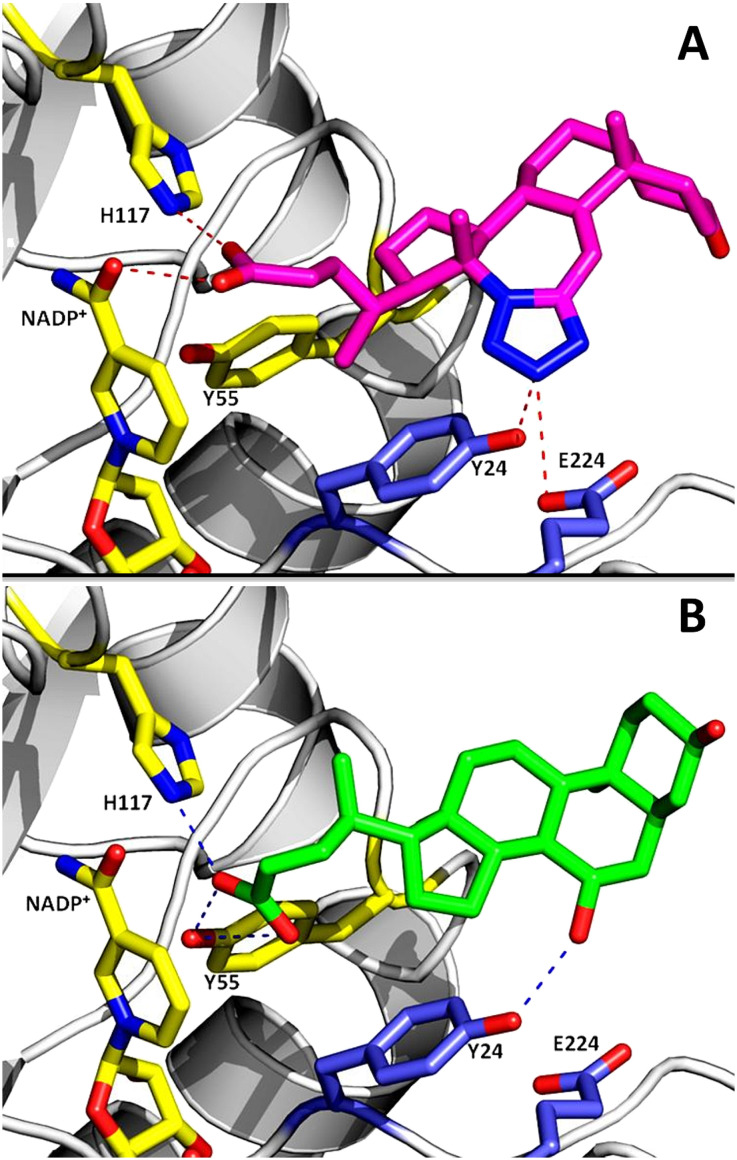
A) Molecular docking of compound 1 (magenta sticks) onto the active site of AKR1C2. Residues involved in hydrogen bonding to 1 are shown as sticks. Catalytic residues (H117, Y55) and the NADP^+^ cofactor are shown as yellow sticks. Hydrogen bonds are shown as dashed lines. For comparison, in panel B) the crystal structure of UDCA (green sticks) in complex with AKR1C2 is shown (PDB 1IHI).

Because compound 1 inhibits both AKR1C2 (86%) and AKR1C3 (88.4%) isoforms, we attempted to predict the molecular basis of AKR1C2 inhibition by 1 using molecular docking. Docking simulations were conducted in Autodock Vina.^49^ The structure of AKR1C2 in complex with a bile acid inhibitor, ursodeoxycholate (UDCA), was chosen as ‘receptor’ because UDCA shares chemically similarity to compound 1.^24^ As a control, UDCA was re-docked onto an apo structure of AKR1C2·NADP^+^ following removal of the coordinates for UDCA in a text editor. Re-docking of UDCA in Autodock Vina was able to reproduce the experimental structure of AKR1C2·NADP^+^·UDCA with identical geometry and a mean docking energy of −9.4 kcal mol^−1^. This docking model for AKR1C2 has been validated for identification of other bile acid inhibitors of AKR1C2.^26^ Under these conditions, a favorable docking energy of −9.0 kcal mol^−1^ was obtained for compound 1. Compound 1 is predicted to form hydrogen bonds with the catalytic site (H117, NADP^+^ cofactor) and, *via* the tetrazole group, with residues in the SP3 binding pocket (Y24), as well as nearby E224. The mode of binding is similar to UDCA, which also forms hydrogen bonds with the catalytic site (Y55, H117) and Y24, but not E224 (Fig. 10).

The binding site for compound 1 on AKR1C2 identified by docking is different than that predicted for AKR1C3 based on the structure of AKR1C3·NADP^+^·(2). AKR1C2 and AKR1C3 are closely-related isoforms, with 91% sequence similarity and 87% identity at the amino acid level (ESI† 1). Based on sequence alignment there are two important differences between AKR1C2 and AKR1C3 isoforms. In the loop consisting of residues 303–315, there are 8 amino acid differences, and three serine residues (S129, S308 and S310) involved in binding compound 2 in AKR1C3 are larger hydrophobic residues in AKR1C2 (I129, L308 and I310) (ESI† 1). These substitutions change the size and hydrophobicity of the AKR1C2 ligand binding pocket, and could explain the differences in binding geometry predicted by docking (ESI† 2). Comparisons of structures of AKR1C3 (PDB 3R7M) and AKR1C2 (PDB 4JQ2) in complex with sulindac support this: sulindac binds in SP2 in AKR1C3 (Fig. 8), but is also rotated toward SP3 in complex with AKR1C2 (ESI† 2).^20^

## Conclusions

Based on results from enzyme activity assays *in vitro*, bile acid derivatives with a C-ring fused tetrazole were identified as strong AKR1C3 inhibitors. Fusion of the tetrazole at the C-ring is important for AKR1C3 inhibitory activity, since B-ring fused tetrazoles were not able to inhibit AKR1C3 activity. A C24 bile acid with a C-ring fused tetrazole (2) was found to inhibit AKR1C3 with an IC_50_ value (∼7 μM) comparable to that reported for pan-AKR1C inhibitor ibuprofen (IC_50_ ∼ 10 μM). Off target co-inhibition of AKR1C2 should be avoided in the context of prostate cancer, since AKR1C2 reduces the potent androgen, 5α-dihydrotestosterone.^4^ Similarly, off-target activation of ERα or AR is a particular concern for design of anticancer compounds based on steroidal scaffolds. Compound 2 was found to display selectivity for AKR1C3 over AKR1C2 and was shown to have no off-target affinity for ERα or AR receptors. Structure determination of AKR1C3 in complex with (2) by protein X-ray crystallography suggests a binding mechanism involving hydrophobic contacts between the steroidal core and residues comprising a subpocket of the AKR1C3 ligand binding site known as SP2. Based on the crystal structure, we propose a mechanism whereby the C24 carboxylate group anchors the inhibitor to the catalytically important oxyanion site, in proximity to the NADPH cofactor, while the C-ring fused tetrazole interacts strongly with the conserved ‘gatekeeper’ tryptophan residue (W227) in the steroid channel of AKR1C3. Comparison with other AKR1C3 structures suggests that (2) inhibits AKR1C3 *via* a mechanism similar to other carboxylate containing inhibitors, such as ibuprofen and sulindac; meanwhile the steroidal scaffold and tetrazole moiety provide opportunities to improve the specificity and potency of this newly identified class of inhibitors. Molecular docking predicts that all of the top inhibitors identified in the present study (1–4) bind AKR1C3 with identical geometry, and thus could inhibit AKR1C3 by a similar mechanism. Compound 1 also inhibits AKR1C2, and molecular docking predicts a binding geometry similar to UDCA. Based on the present study, bile acids with a C-ring fused tetrazole represent a new class of AKR1C3 inhibitors. Results from the present study should facilitate structure-based design of improved AKR1C3 inhibitors based on bile acid fused tetrazoles.

## Materials and methods

### Chemicals

#### Compounds 1–8

Compounds 1–8 were characterized by ^1^H and ^13^C NMR spectroscopy, IR spectroscopy and HRMS, and their chemical synthesis and full characterization have been published.^28^

##### Compound 1

12*a*-Aza-3α-hydroxy-12*a*-homo-tetrazolo[5′,1′:12,12*a*]-5β-chol-9(11)-en-24-oic acid

##### Compound 2

12*a*-Aza-3α-hydroxy-12*a*-homo-tetrazolo[5′,1′:12,12*a*]-5β-cholan-24-oic acid

##### Compound 3

12*a*-Aza-3α,7α-dihydroxy-12*a*-homo-tetrazolo[5′,1′:12,12*a*]-5β-chol-9(11)-en-24-oic acid

##### Compound 4

12*a*-Aza-3α,7α-dihydroxy-12*a*-homo-tetrazolo[5′,1′:12,12*a*]-5β-cholan-24-oic acid

##### Compound 5

Methyl 7*a*-aza-3α,12α-diacetoxy-7*a*-homo-tetrazolo[5′,1′:7,7*a*]-5β-cholan-24-oate

##### Compound 6

Ethyl 3α-acetoxy-7*a*-aza-7*a*-homo-tetrazolo[5′,1′:7,7*a*]-5β-cholan-24-oate

##### Compound 7

7*a*-Aza-3α,12α-dihydroxy-7*a*-homo-tetrazolo[5′,1′:7,7*a*]-5β-cholan-24-oic acid

##### Compound 8

7*a*-Aza-3α-hydroxy-7*a*-homo-tetrazolo[5′,1′:7,7*a*]-5β-cholan-24-oic acid

### Expression and purification of recombinant human AKR1C2 and AKR1C3

Expression and purification of AKR1C2 and AKR1C3 isoforms was performed as described previously.^26,27,50^ Plasmid DNA encoding 6×His-tagged human AKR1C2 and AKR1C3 in pET28b(+) expression vectors were obtained from Professor Dr. Chris Bunce (University of Birmingham, UK) for expression in *E. coli*.^11^ Competent BL21 (DE3) *E. coli* cells were transformed with pET28-AKR1C2 or pET28-AKR1C3 and grown at 37 °C in LB media supplemented with kanamycin (50 μg ml^−1^). AKR1C expression was induced at a cell density of OD_600_ = 0.6 with 1 mM IPTG (isopropyl β-d-1-thiogalactopyranoside). Induced cells were grown for 6 hours before harvesting by centrifugation. Cell pellets were resuspended in buffer A (50 mM sodium phosphate, 500 mM NaCl, pH 8.0) supplemented with lysozyme (1 mg ml^−1^) and cells lysed by three freeze–thaw cycles followed by sonication. The cell lysate was clarified by centrifugation at 12 000×*g* for 45 minutes at 4 °C. The clarified supernatant was applied to a 1 ml HisTrap column packed with nickel sepharose (GE healthcare) and equilibrated in 10 column volume (CV) buffer A. The column was washed in 10 CV buffer A, followed by 10 CV buffer A with 40 mM imidazole. AKR1C isoforms were eluted in 5 CV Buffer A with 400 mM imidazole. Pooled fractions containing AKR1C2 or AKR1C3 were exchanged into buffer B (10 mM potassium phosphate, 10 mM NaCl, pH 7.0, supplemented with 1.2 mM NADP^+^) by dialysis using a 10 kDa molecular weight cut-off (Slide-a-lyzer, ThermoFisher). After dialysis, the protein sample was subjected to centrifugation at 14 000×*g* at 4 °C and applied to a gel filtration column. Proteins were purified by gel filtration chromatography using a 120 ml HiLoad Superdex 200 pg column (Sigma), equilibrated in buffer B using an Äkta Basic FPLC system (GE Healthcare). Protein purity was monitored by SDS-PAGE with Coomassie staining and protein concentration was quantified by the Bradford colorimetric assay and by monitoring absorption at 280 nm by UV spectroscopy. Purified AKR1C2 and AKR1C3 were then concentrated to 50 mg ml^−1^ (AKR1C3) or 40 mg ml^−1^ (AKR1C2) by centrifugation at 14 000×*g* and 4 °C using an Amicon spin column with a molecular weight cut-off of 15 kDa (Millipore). Purified proteins were stored at −80 °C in 10 mM potassium phosphate, 10 mM NaCl, pH 7.0, supplemented with 2 mM NADP^+^ (Sigma) and 30% glycerol.

### Measurement of AKR1C3 enzyme activity by NADPH fluorescence spectroscopy

The enzymatic activity of AKR1C3 was measured by monitoring the change in NADPH fluorescence during substrate reduction. Reactions were conducted at 37 °C in 100 mM potassium phosphate buffer pH 6.0 with 0.25 mM NADPH and 16.7 μM 9,10-phenanthrenequinone (PQ, a pan-AKR1C substrate). AKR1C3 concentration was held constant at 80 μg ml^−1^ (∼2 μM). The effect of compounds 1–8 on the ability of AKR1C3 to reduce PQ was tested at a final concentration of 33.3 μM in 96-well microplates (Greiner bio-one). All the compounds were dissolved in DMSO (Sigma), and the final DMSO concentration in each reaction was 2%. Reactions were initiated by addition of enzyme and NADPH fluorescence was monitored at an emission wavelength of 460 nm (excitation at 340 nm) for 30 minutes at 30 second intervals. To obtain percent inhibition, normalized NADPH fluorescence at 460 nm was plotted against reaction time and the resulting slope was calculated. The slope obtained for PQ-only control reactions was defined to represent 100% enzymatic activity. Blank reactions were measured in the absence of enzyme for normalization of the data. The percent inhibition (% inhibition) for each test compound was then obtained as:% inhibition (compound) = 100 − [slope (PQ + compound)/slope (PQ-only) × 100]where slope (PQ + compound) corresponds to the slope of the reaction in the presence of the test compound, and slope (PQ-only) corresponds to the slope of the reaction without an inhibitor. For compound 2, a dose–response curve for AKR1C3 inhibition was also obtained by measuring the effect of increasing inhibitor concentrations from 0 to 100 μM.

### Measurement of AKR1C2 enzyme activity by NADPH absorbance spectroscopy

The effect of compounds 1 and 2 on the ability of AKR1C2 to reduce 9,10-phenanthrenequinone was measured using a standard assay based on NADPH absorption, as previously described.^26,27^ Reactions were conducted at 36 °C in 100 mM potassium phosphate buffer pH 7.0 with 0.2 mM NADPH and 0.1 μM 9,10-phenanthrenequinone (PQ) as a standard AKR1C substrate. AKR1C2 protein concentration was held constant at 0.1 mM. The effect of compounds 1 or 2 on AKR1C2 activity was tested in triplicate at a final concentration of 40 μM. All the compounds were dissolved in DMSO, and the final DMSO concentration in each reaction was 2%. Reactions were initiated by addition of AKR1C2 enzyme and NADPH absorbance was monitored every 30 seconds at a wavelength of 340 nm for 15 minutes using a Thermo Scientific Multiscan GO spectrophotometer in 96-well microplates (Greiner bio-one). To obtain percent inhibition, the absorbance at 340 nm was plotted against reaction time and the resulting slope was calculated. The slope obtained for PQ-only control reactions was defined to represent 100% enzymatic activity. Blank reactions were measured in the absence of enzyme for normalization of the data. The percent inhibition (% inhibition) for each test compound was then obtained as described above for AKR1C3.

### Crystallization of human AKR1C3 in complex with NADP^+^ and compound 2

Protein expression and purification for crystallization experiments for AKR1C3 were conducted as previously described.^50^ Human AKR1C3 was purified by nickel affinity chromatography and a final step of size exclusion chromatography to remove aggregates. Before crystallization experiments, purified AKR1C3 in buffer B (10 mM potassium phosphate pH 7.0, 1 mM EDTA, 1 mM DTT, supplemented with 1.2 mM NADP^+^) at concentration 50 mg ml^−1^ was clarified by centrifugation at 20 000×*g* at 4 °C. Compound 2, from a 100 mM stock solution dissolved in 100% DMSO, was then added to pure AKR1C3 at a molar ratio of 2 : 1 ligand : protein, at a final concentration of 2.7 mM, followed by incubation on ice for 30 minutes. Following purification, recombinant AKR1C3 contains an N-terminal His6-tag followed by a thrombin recognition site (shown in bold): MGSSHHHHHHSSG**LVPRGS**H encoded by the pET28b(+) vector. Because the N-terminal His6-tag could interfere with crystallization, immediately prior to crystallization trials, bovine thrombin (Sigma) was added at a final concentration of 0.1 mg ml^−1^, for *in situ* proteolysis of the His6-tag during crystallization. Addition of thrombin at this stage of crystallization has been shown to improve AKR1C3 crystal morphology and X-ray diffraction properties.^50^ Crystallization screening was performed at 18 °C by sitting-drop vapor diffusion using Gryphon (Art Robbins Instruments, USA) and Oryx8 (Douglas Instruments, UK) crystallization workstations in 96-well sitting drop MRC 3-well plates (Jena Bioscience, Germany). For crystallization trials, we focused on the MORPHEUS crystallization screen, because the diffraction quality of any resulting crystals can be tested by flash freezing under a nitrogen cryostream without additional screening for cryoprotectants.^51^ A total volume of 300 nl of a mixture of protein with inhibitor compound 2 and thrombin, and precipitant solutions in a 1 : 1 volume ratio was equilibrated against a 30 μl reservoir solution containing the precipitant solution. The crystallization process was monitored using a Minstrel DT UV automated imaging system with a Gallery DT plate hotel (Rigaku, Japan) and an Olympus SZX10 optical microscope (Olympus, Japan). Conditions which yielded crystals were manually optimized by hanging-drop vapor diffusion using 24-well crystallization plates (Hampton Research): hanging drops contained between 1 and 2 μl protein and precipitant at a 1 : 1 volume ratio; a reservoir volume of 500 μl was used. Crystals of AKR1C3 in complex with NADP^+^ and compound 2 were grown in Morpheus condition H12 corresponding to 12.5% w/v PEG 1000, 12.5% w/v PEG 3350, 12.5% v/v MPD and 0.02 M each of dl-glutamic acid monohydrate, dl-alanine, glycine, dl-lysine monohydrochloride, and dl-serine; in 0.1 M bicine/Trizma base, pH 8.5.^51^ Crystals appeared overnight and grew to a final size of ∼40 × 20 × 20 μm after 4 days, before harvesting without additional cryoprotection.^51^

### X-ray diffraction data collection and processing

The diffraction properties of AKR1C3·NADP^+^·(2) crystals were tested using an in-house system before being transfer to a synchrotron (MX 14.1 operated by the Joint Berlin MX-Laboratory at the BESSY II electron-storage ring) for data collection.^52,53^ Diffraction quality was tested at 100 K on an in-house MicroMax-007 HF Microfocus X-ray generator with a VariMax VHF Arc Sec confocal optical system (Rigaku, Japan) equipped with an AFC11 partial χ four-axis goniometer (Rigaku, Japan), a PILATUS 300K detector (Dectris, Switzerland) and a Cryostream 800 cryocooling system (Oxford Cryosystems, England). Diffraction data was then collected at MX 14.1 operated by the Joint Berlin MX-Laboratory at the BESSY II electron-storage ring in Berlin-Adlershof, Germany.^52,53^ Diffraction data were processed using XDS.^54^ Crystal parameters and data collection statistics are listed in Table 2.

### Structure determination of AKR1C3 in complex with compound 2

X-ray diffraction data was collected to a resolution of 1.40 Å, and analysis of the data revealed a *P*1 space group with two monomers in the asymmetric unit. Initial phases for structure determination of human AKR1C3 in complex with compound 2 were obtained by molecular replacement using the program PHASER in CCP4.^55,56^ The structure of human AKR1C3 in complex with a steroidal inhibitor was used as the search model (PDB entry 1ZQ5).^21,55,56^ Resulting *F*_o_ − *F*_c_ difference electron density maps were then analyzed for electron density corresponding to compound 2. Clear electron density for compound 2 was visible following molecular replacement and initial refinement (see Fig. 5). Model refinement was performed using REFMAC5 (ref. 57) in the CCP4 suite^56^ coupled with manual model building and real space refinement in the program COOT.^56–58^ For early rounds of REFMAC refinement, local non-crystallographic symmetry (NCS) restraints and translation, rotation, and screw-rotation (TLS) refinement were used.^59^ Local NCS restraints were defined in REFMAC between chain A (residues 6–323) and chain B (residues 6–323). Two TLS groups were also defined, consisting of chain A (TLS group 1, residues 6 to 323) and chain B (TLS group 2, residues 6 to 323). NCS restraints and TLS refinement were not used in final refinement steps. Iterative cycles of refinement and model building were then carried out using the programs REFMAC and COOT.^57,58^ Coordinates for compound 2 were added during the final stages of refinement using tools available in COOT.^58^ Bound water molecules were added manually and using the automated water picking routines available in COOT and REFMAC, followed by visual inspection of the hydrogen bonding environment. The MolProbity server was used for evaluation of the final model quality.^40^ Structure refinement statistics are listed in Table 2. All figures representing structures were created using PyMOL (The PyMOL Molecular Graphics System, Version 0.99, Schrödinger, LLC). Atomic coordinates and structure factors were deposited in the PDB under the accession code 8BBS.

### Fluorescence assay for receptor binding in yeast cells

For the assay, the following yeast strains and plasmids were used as described, provided by Dr. Blake Peterson, University of Kansas: FY250 (MATα, *ura3-52*, *his32Δ00*, *leu2Δ1*, *trp1Δ6*) and pRF4-6-AR LBD-EYFP.^33^ Yeast was transformed using a standard lithium acetate/polyethylene glycol procedure.^60^ Yeast cells transformed with plasmid were selected on tryptophan dropout agar plates following 3 days of growth at 30 °C. Master plates were created from individual clones and stored at 4 °C. The fluorescence assay in yeast was optimized for assessment of the relative binding affinities of steroidal derivatives for estrogen receptor α (ERα), ERβ and the androgen receptor (AR). Selective medium supplemented with 2% raffinose was inoculated with recombinant yeast cells and incubated until saturation in a Biosan orbital shaker-incubator ES-20/60. The optical density of cell cultures was measured spectroscopically at 600 nm (OD_600nm_) using a Nicolet Evolution 100 UV-vis spectrophotometer. Saturated pre-cultures were diluted at OD_600nm_ ∼ 0.1 in fresh medium and incubated at 30 °C for 2–3 generations. Expression of ERα- ERβ- or AR-LBD-YFP proteins was induced in mid-log phase (OD_600nm_ ∼ 0.4–0.6) by addition of galactose (2% final concentration). Test compounds 1–8, estrone (negative control AR ligand, positive control ER ligand) or androstenedione (positive control AR ligand, negative control ER ligand) were then added at a final concentration of 10 μM. Treated cells were incubated at room temperature for 15 h in the dark. Fluorescence measurements were conducted in triplicate in 96-well plates (Greiner bio-one) using 150 μL of cell suspensions. Growth medium served as a blank. Fluorescence intensity was recorded using a Fluoroskan Ascent FL fluorimeter with excitation and emission wavelengths of 485 and 538 nm, respectively. For signal normalization, fluorescence intensity was divided by optical density at 600 nm. Ligand binding affinity was expressed as fold fluorescence against the negative control. Histograms were generated using Origin Pro 8 software. Error bars represent propagated standard errors of the mean. For visualization of individual cell fluorescence distribution and qualitative analysis 3 μl of concentrated cell suspension was observed under a fluorescence microscope Olympus BX51 using a FITC filter.

## Molecular docking

### Preparation of AKR1C3 receptor coordinates

Coordinates from the structure of AKR1C3 in complex with NADP^+^ and compound 2 (PDB 8BBS) were used as ‘receptor’ for molecular docking simulations. Ligands and bound water molecules were removed using a text editor, while the NADP^+^ cofactor was retained. Hydrogen atoms were added to the receptor and Gasteiger partial charges were assigned using the script ‘receptor.c’ in the program VEGAZZ 3.1.0.^61^ Non-polar hydrogen atoms were merged and receptor coordinate files were converted to PDBQT format for molecular docking simulations in Autodock 4.2.

### Preparation of ligand coordinates

Based on the 3D structure of compound 2, structural models for test compounds 1–4 were created in the program Avogadro 1.1.2, an open-source molecular builder and visualization tool (https://avogadro.cc/).^62^ Hydrogen atoms were added and geometry optimized using an MMF94s force field with 500 steps of conjugate gradient energy minimization and 500 steps of steepest descent energy minimization with a convergence setting of 10 × 10^−7^. Gasteiger partial charges were assigned and nonpolar hydrogen atoms were merged using the script ‘ligand.c’ in VEGAZZ 3.1.0 to create PDBQT files for molecular docking simulations in Autodock 4.2.^61^

### Molecular docking simulations in Autodock 4.2

Molecular docking screening was conducted in Autodock 4.2 using the Lamarckian genetic algorithm, with Grid maps calculated using AutoGrid 4 in the PyRx virtual screening tool.^45,46^ Grid maps were centered on the coordinates of (2) present in the AKR1C3·NADP^+^·(2) complex (gridcenter *x* = −15.159, *y* = 27.616, *z* = −9.818), using a grid size of 50 × 50 × 50 Å and a default grid spacing of 0.375 Å. Grid maps were calculated for all atom types present in compounds 1–4, while electrostatic and desolvation maps were created using the default dielectric value of −0.1465. Initial ligand coordinates, orientation and dihedral offset values were random. The number of torsional degrees of freedom for test compounds was determined during the ligand preparation stage. AutoGrid and AutoDock calculations were conducted using the PyRx virtual screening tool.^46^ The number of energy evaluations was set to the default value of 250 000 with a GA population size of 150. A total of 10 hybrid GA-LS (hybrid genetic algorithm-local search) runs were performed for each docking simulation. Control redocking simulations were conducted using compound 2 as the ligand. Results were visualized using the open-source program PyMOL v0.99 (ref. 44) and compared with the structure of AKR1C3 in complex with NADP^+^ and compound 2.

### Molecular docking simulations in Autodock Vina

Molecular docking of compound 1 onto the structure of AKR1C2 in complex with UDCA (PDB 1IHI)^24^ was conducted using Autodock Vina and the PyRx screening tool as described,^26,46,49^ with the following parameters: exhaustiveness = 100, centered at *x* = −1.3723, *y* = 32.0891 and *z* = 36.9620 using a search space of 25 × 25 × 25 Å. Results were visualized in PyMOL v0.99 and compared to the structure of AKR1C2 in complex with UDCA (PDB 1IHI).

## Conflicts of interest

There is no conflict of interest to declare.

## Supplementary Material

MD-014-D2MD00387B-s001

MD-014-D2MD00387B-s002
