# Supplementary material for: X-ray structure of human aldo–keto reductase 1C3 in complex with a bile acid fused tetrazole inhibitor: experimental validation, molecular docking and structural analysis
Source: RSC Med Chem. 2022 Dec 1;14(2):341–55. doi: 10.1039/d2md00387b (PMC9945864; doi:10.1039/d2md00387b)
Supplement: MD-014-D2MD00387B-s001 [file MD-014-D2MD00387B-s001.pdf]

Electronic Supplementary Material (ESI) for RSC Medicinal Chemistry.
This journal is © The Royal Society of Chemistry 2022

```
                   10        20        30        40        50
                    |         |         |         |         |
AKR1C2   1     MDSKYQCVKLNDGHFMPVLGFGTYAPAEVPKSKALEAVKLAIEAGFHHIDSAHVYNNEEQ  60
               MDSK+QCVKLNDGHFMPVLGFGTYAP EVP+SKALE  KLAIEAGF HIDSAH+YNNEEQ
AKR1C3   1     MDSKHQCVKLNDGHFMPVLGFGTYAPPEVPRSKALEVTKLAIEAGFRHIDSAHLYNNEEQ  60
                    |         |         |         |         |         |
AKR1C2   61    VGLAIRSKIADGSVKREDIFYTSKLWSNSHRPELVRPALERSLKNLQLDYVDLYLIHFPV  120
               VGLAIRSKIADGSVKREDIFYTSKLWS  HRPELVRPALE SLK  QLDYVDLYLIH P+
AKR1C3   61    VGLAIRSKIADGSVKREDIFYTSKLWSTFHRPELVRPALENSLKKAQLDYVDLYLIHSPM  120
                    |         |         |         |         |         |
AKR1C2   121   SVKPGEEVIPKDENGKILFDTVDLCATWEAMEKCKDAGLAKSIGVSNFNHRLLEMILNKP  180
               S+KPGEE+ P DENGK++FD VDLC TWEAMEKCKDAGLAKSIGVSNFN R LEMILNKP
AKR1C3   121   SLKPGEELSPTDENGKVIFDIVDLCTTWEAMEKCKDAGLAKSIGVSNFNRRQLEMILNKP  180
                    |         |         |         |         |         |
AKR1C2   181   GLKYKPVCNQVECHPYFNQRKLLDFCKSKDIVLVAYSALGSHREEPWVDPNSPVLLEDPV  240
               GLKYKPVCNQVECHPYFN+ KLLDFCKSKDIVLVAYSALGS R++ WVDPNSPVLLEDPV
AKR1C3   181   GLKYKPVCNQVECHPYFNRSKLLDFCKSKDIVLVAYSALGSQRDKRWVDPNSPVLLEDPV  240
                    |         |         |         |         |         |
AKR1C2   241   LCALAKKHKRTPALIALRYQLQRGVVVLAKSYNEQRIRQNVQVFEFQLTSEEMKAIDGLN  300
               LCALAKKHKRTPALIALRYQLQRGVVVLAKSYNEQRIRQNVQVFEFQLT+E+MKAIDGL+
AKR1C3   241   LCALAKKHKRTPALIALRYQLQRGVVVLAKSYNEQRIRQNVQVFEFQLTAEDMKAIDGLD  300
                    |         |
AKR1C2   301   RNVRYLTLDIFAGPPNYPFSDEY  323
               RN+ Y   D FA  PNYP+SDEY
AKR1C3   301   RNLHYFNSDSFASHPNYPYSDEY  323
```

Supplemental. Sequence alignment of human AKR1C2 and AKR1C3 using the 'align two or more sequences' feature of protein BLAST (https://blast.ncbi.nlm.nih.gov). AKR1C3 shares 91% sequence similarity and 87% identity with AKR1C2. Identical residues are shown in the consensus sequence between the two compared sequences. Similar residues are marked with a '+'. Residues in contact with compound **2** in the structure of **AKR1C3·NADP+·2** are shown in bold and highlighted in yellow. Of these 11 residues, 3 residues that are different in AKR1C2 are shown in red and highlighted in yellow.
