# Supplementary material for: X-ray structure of human aldo–keto reductase 1C3 in complex with a bile acid fused tetrazole inhibitor: experimental validation, molecular docking and structural analysis
Source: RSC Med Chem. 2022 Dec 1;14(2):341–55. doi: 10.1039/d2md00387b (PMC9945864; doi:10.1039/d2md00387b)
Supplement: MD-014-D2MD00387B-s002 [file MD-014-D2MD00387B-s002.pdf]

Electronic Supplementary Material (ESI) for RSC Medicinal Chemistry.
This journal is © The Royal Society of Chemistry 2022

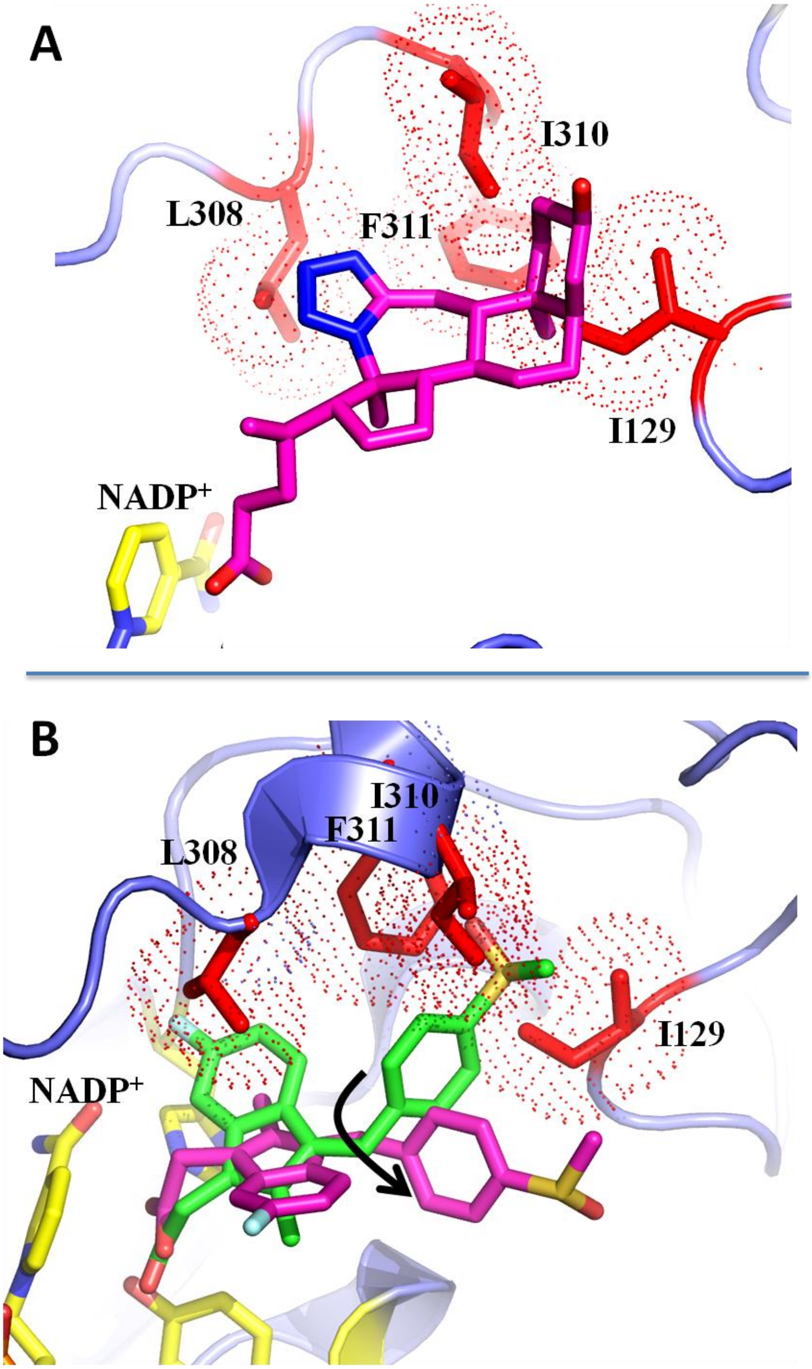

Supplemental. **A)** Superposition of AKR1C3 in complex with compound **2** onto coordinates for AKR1C2 in complex with ursodeoxycholate (PDB 1IHI), using the align function in PyMol. Only compound **2** and AKR1C2 are shown. AKR1C2 residues that would visibly clash with compound **2** are shown as red sticks with van der Waals radii shown as red dots. **B)** Superposition of AKR1C3 (PDB 3R7M) and AKR1C2 (PDB 4JQ2) in complex with sulindac . The positions of sulindac bound to AKR1C3 (green sticks) and AKR1C2 (magenta sticks) are compared against coordinates for AKR1C2. AKR1C2 residues that would visibly clash with sulindac in the AKR1C3 position are shown as red sticks with van der Waals radii shown as red dots.
